# Automated Signal Quality Assessment of Single-Lead ECG Recordings for Early Detection of Silent Atrial Fibrillation

**DOI:** 10.3390/s23125618

**Published:** 2023-06-15

**Authors:** Markus Lueken, Michael Gramlich, Steffen Leonhardt, Nikolaus Marx, Matthias D. Zink

**Affiliations:** 1 Medical Information Technology (MedIT), Helmholtz-Institute for Biomedical Engineering, RWTH Aachen University, 52074 Aachen, Germany; 2 Department of Internal Medicine I-Cardiology, University Hospital RWTH, 52074 Aachen, Germany

**Keywords:** atrial fibrillation, ECG signal quality assessment, single-lead ECG screening

## Abstract

Atrial fibrillation (AF) is an arrhythmic cardiac disorder with a high and increasing prevalence in aging societies, which is associated with a risk for stroke and heart failure. However, early detection of onset AF can become cumbersome since it often manifests in an asymptomatic and paroxysmal nature, also known as silent AF. Large-scale screenings can help identifying silent AF and allow for early treatment to prevent more severe implications. In this work, we present a machine learning-based algorithm for assessing signal quality of hand-held diagnostic ECG devices to prevent misclassification due to insufficient signal quality. A large-scale community pharmacy-based screening study was conducted on 7295 older subjects to investigate the performance of a single-lead ECG device to detect silent AF. Classification (normal sinus rhythm or AF) of the ECG recordings was initially performed automatically by an internal on-chip algorithm. The signal quality of each recording was assessed by clinical experts and used as a reference for the training process. Signal processing stages were explicitly adapted to the individual electrode characteristics of the ECG device since its recordings differ from conventional ECG tracings. With respect to the clinical expert ratings, the artificial intelligence-based signal quality assessment (AISQA) index yielded strong correlation of 0.75 during validation and high correlation of 0.60 during testing. Our results suggest that large-scale screenings of older subjects would greatly benefit from an automated signal quality assessment to repeat measurements if applicable, suggest additional human overread and reduce automated misclassifications.

## 1. Introduction

Atrial fibrillation (AF) is a cardiac arrhythmia with a high and increasing prevalence in aging societies, which is associated with a risk for stroke and heart failure. AF affects around 1–2% of the overall population [1,2]. However, the prevalence of AF increases with age, with one European study reporting a prevalence of 0.7% in the age group of 55–59 years and 17.8% in individuals aged over 85 years [3]. AF is associated with a 5-fold increased risk for strokes [4], a 3-fold increased risk for heart failure [5], and an overall higher risk for mortality [5]. Additionally, untreated AF appears to be associated with a 50% increased risk for dementia [6].

The European Society of Cardiology (ESC) Guidelines for the management of atrial fibrillation [7] describe a progressive character of AF if untreated, which often starts as paroxysmal AF and evolves to persistent AF and long-standing/permanent AF. Symptomatic or asymptomatic episodes may appear in any of these stages. A diagnosis of AF is typically established through a 12-lead ECG in patients with suspected but undocumented AF. However, early detection of onset AF can become cumbersome since it often manifests in an asymptomatic and paroxysmal nature [8], which is known as silent AF, and AF can only be diagnosed if present during an ECG recording. Therefore, such patients may remain undiagnosed or may only be diagnosed incidental when being treated for other conditions.

Given the high prevalence of silent AF of up to 30% in patients with risk for stroke, the US National Heart, Lung, and Blood Institute (NLBHI) recommends the use of noninvasive methods to identify and characterize incident and recurrent AF, especially in clinical and research settings [9]. Recently, various devices for AF detection, such as stand-alone or mobile systems, have become widely available for large-scale investigations [10]. These include electrocardiographic, photoplethysmographic or mechanical sensors with varying levels of accuracy for automated AF detection, whereas ECG sensors generally provide higher accuracy [11,12]. Machine learning and artificial intelligence-assisted diagnostics are expected to significantly improve the specificity and sensitivity of large-scale screenings when trained on a large set of data sets that will become available in the near future [13].

Large-scale ECG measurement devices, such as ECG hand-held sticks, rely on easy-to-apply electrode conditions and signal acquisition to provide accurate recordings of cardiac electrical activity. For reasons of simplicity, these measurement devices typically realize a single-lead measurement to capture the cardiac electrical signals [10]. The electrode characteristics of these devices are critical for accurate signal acquisition, as they must have low impedance and high signal-to-noise ratio (SNR) to capture small amplitude signals [14]. Signal quality can be affected by various factors, such as patient movement, electrode-skin interface characteristics, and environmental noise [15]. Motion artifacts may distort the ECG signal, leading to false positive or false negative decisions in AF detection [16].

The accuracy of automated AF detection is limited [17] and preliminary results indicate that it may be associated with age and underlying comorbidities, which are also the target groups for AF screening [18]. A reliable signal quality assessment (SQA) is of major importance to optimize the classification performance independently of individual influences. SQA for ECG recordings has been proposed in the literature before using different approaches. Hayn et al. presented an algorithm, which assess signal quality base on the QRS detection performance [19]. They implemented a threshold-based classification based on four different signal features. Li implemented an SQA method based on spectral analysis, which can assess the signal quality of a single-lead ECG quantitatively [20]. Zhang and Zhao described an effective system for ECG quality assessment based on simple heuristic fusion and fuzzy comprehensive evaluation of the signal quality indexes, which can discriminate between high- and poor-quality ECGs [21]. Their algorithmic approach was based on hard thresholding of two spectral features and one statistical parameter. Clifford et al. proposed an algorithm based on novel and previously published signal quality metrics, which can achieve a high classification accuracy for determining the clinical acceptability of ECGs [15].

Recent research has increasingly focused on the accuracy of wearable devices, particularly wrist-worn devices and hand-helds, to detect silent and paroxysmal AF. Belani et al. found that wrist-worn devices, including Apple Watch, Samsung, and KardiaBand, demonstrated promising results in detecting AF in patients with paroxysmal AF [12]. Prasitlumkum et al. found that smartphones and smartwatches had similar diagnostic accuracies in detecting AF, with a sensitivity of 94% and a specificity of 96% for smartphones and a sensitivity of 93% and a specificity of 94% for smartwatches [22]. Zink et al. used an single-lead ECG stick to detect AF within a large-scale pharmacy-based study [23]. Pereira et al. reviewed the state of the art in AF detection with wrist-worn devices and found that the methods show excellent accuracy for AF detection, but more studies are needed to determine the accuracy of the technology for ambulatory long-term monitoring [24].

This study aimed to determine the accuracy of a single-lead ECG device in a pharmacy-based screening approach using a MyDiagnostick ECG device. A machine learning approach based on multiple features from different domains is applied to provide an *artificial intelligence-based signal quality assessment* (AISQA) to support the reliability of the automated AF screening. The rest of this paper is organized as follows. In Section 2, we analyze the basic requirements associated with the ECG sensor setup used and discuss the limitations and possibilities of such devices. In Section 3, we present an approach to quantify the signal quality of ECG recordings obtained in a large-scale clinical study. The results are presented and discussed in Section 4 and Section 5, respectively. Finally, we conclude this paper with Section 6 and give an outlook on the next steps in this project.

## 2. Analysis of Prerequisites

### 2.1. Single-Lead ECG Device for AF Detection

**Figure 1:**
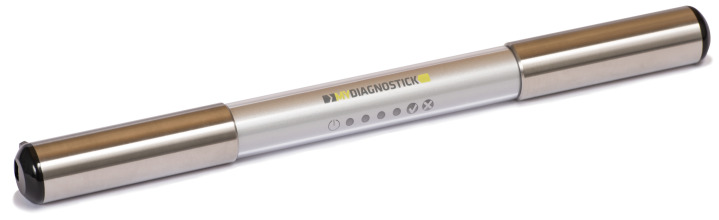
The MyDiagnostick device used in the our study. The device incorporates two metal handle electrodes and performs an Einthoven I-lead ECG when held by the user.

In this study, a hand-held device (MyDiagnostick, Applied Biomedical Systems, Maastricht, The Netherlands) was used to record ECG data on a large-scale basis. The device is depicted in Figure 1. It is designed to be used by healthcare professionals for easy diagnosis of AF. The device has flat metal electrodes at both ends that are grasped by the subjects to obtain the Einthoven I-lead ECG signal with a sampling rate of $f_{s}=200\;\mathrm{Hz}$. The ECG signal is then analyzed by the internal software to detect any abnormalities or AF-related arrhythmia. The system can be connected to a computer or mobile device for real-time monitoring and offline data analysis. The device has been shown to be a reliable and effective tool for the diagnosis of arrhythmia, with a high level of agreement with standard 12-lead ECG measurements.

### 2.2. Characteristics and Simulation of Metal Electrodes and Measurement Device

In order to implement sophisticated algorithms for assessing the signal quality and finally classifying for different cardiac arrhythmia, the signal morphology resulting from the environmental conditions and the measurement device itself needs to be understood in detail. Along with the metal electrodes of the utilized device and the specific measurement setup, some specific characteristics of the signal processing chain arise, which need further consideration. First, metal electrodes are not adhesively connected to the skin and, therefore, prone to an increased influence of motion artifacts. Additionally, gel-based wet-contact electrodes (usually Ag/AgCl) provide an optimal interface to the ionic conduction over the skin-electrode interface [25]. The main difference between these two types of electrodes is the categorization into polarizable and non-polarizable electrodes, respectively. In polarizable electrodes, the metal electrode material cannot ionize into the salt solution and they are therefore prone to charge separation caused by the increased capacitive properties due to a possibly oxidized electrode surface [14].

**Figure 2:**
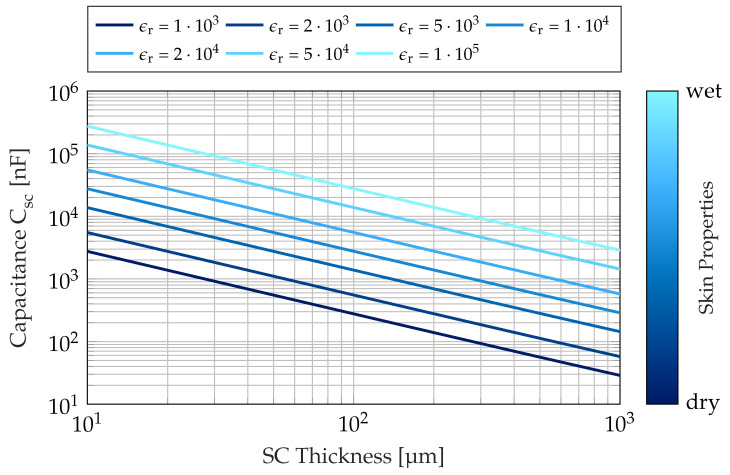
Capacitance of the stratum corneum over the layer thickness. The relative permittivity is additionally dependent on the skin humidity.

Additionally, metal electrodes highly depend on current characteristics of the connecting skin area and underlying tissue properties. Especially when being applied to the hands, the outer layer of the skin, the *stratum corneum* (SC), adds a significant dynamically changing impact on the overall skin-electrode impedance characteristics. The complex impedance can be modeled by a capacitance and a parallel resistance. The resistance of the SC $R_{\mathrm{sc}}$ varies between several 100kΩ and 10MΩ [26]. The capacitance $C_{\mathrm{sc}}$ of the SC was approximated by a cylindrical capacitance representation, which can be described by

(1) $$C_{\mathrm{sc}}=2\;\pi \;\epsilon_{0}\epsilon_{r}\;\frac{l_{\mathrm{elec}}}{\ln\left(\frac{d_{\mathrm{elec}}+d_{\mathrm{sc}}}{d_{\mathrm{elec}}}\right)},$$

where $\epsilon_{0}$ denotes the vacuum permittivity, $l_{\mathrm{elec}}$ and $d_{\mathrm{elec}}$ are the length and the radius of the metal handle electrodes, respectively, and $d_{\mathrm{sc}}$ is the thickness of the SC of the palm. The relative permittivity $\epsilon_{r}$ of the SC was reported by Gabriel et al. [27]. It can be regarded as approximately constant over the range of frequencies that are of interest to ECG analysis and varies in magnitude from $10^{3}$ to $10^{5}$ with the respective humidity conditions of the skin [27,28]. The thickness $d_{\mathrm{sc}}$ of the SC on the hands strongly depends on individual patient characteristics and location and is typically in the range of several 10µm up to 800µm [29,30]. The capacity of the SC was calculated for an effective electrode length of $l_{\mathrm{elec}}=4.5\;\mathrm{cm}$ and an electrode radius of $d_{\mathrm{elec}}=1.1\;\mathrm{cm}$ according to the device manual. The results presented in Figure 2 suggest a capacity range of 10nF to 100µF over the range of humidity conditions and SC thickness.

**Figure 3:**
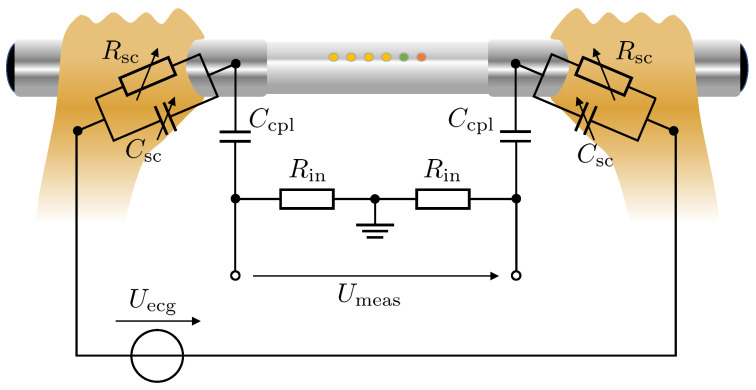
Simplified equivalent circuit diagram of the ECG measurement chain provided by the device used in this study. The circuit consists of the internal voltage source $U_{\mathrm{ecg}}$, which equals a standard ECG signal measurement, the skin-electrode interface, $C_{\mathrm{sc}}$ and $R_{\mathrm{sc}}$, of the metal electrodes, the input high pass-filter characteristic formed by the coupling capacity $C_{\mathrm{cpl}}$ and the input impedance $R_{\mathrm{in}}$ of the analog amplifiers.

Besides the additional influences of the skin-electrode interface in this specific application and characteristics of the polarizable nature of stainless steel electrodes, systems like this incorporating metal electrodes often include actively-driven buffers with high input impedance $R_{\mathrm{in}}$ and coupling capacities $C_{\mathrm{cpl}}$. In this case, the coupling capacity and the input resistance or bias resistance form a passive high-pass filter with cut-off frequencies in the lower 1Hz range to suppress baseline wandering. This methodology additionally affects the signal morphology but, on the other hand, provides a more stable measurement signal. The equivalent circuit diagram is presented in Figure 3. To evaluate the influence of the previously described elements on the respective ECG signal morphology, the electrical model was simulated in MATLAB R2021b Simscape.

In our experiments, the input voltage $U_{\mathrm{ecg}}$ to the system was provided by an artificially generated ECG signal that was given by the synthesizer framework of Hoog Antink et al. [31]. The ECG signal prototypes of the synthesizer are based on templates extracted from real Einthoven I-lead ECG signals provided by a publicly available database [32]. Prior to each simulation run, an ECG signal of 300s length was generated, as would be expected when recording with a conventional patient monitor and standard ECG electrodes. Besides the correlation of the template morphology to a real ECG, the input signal additionally corresponded to the amplitude- and frequency-modulated characteristics of a realistic ECG signal to also capture dynamical inter-beat variabilities. The measured voltage $U_{\mathrm{meas}}$ corresponds to the voltage at the input of the instrumentation amplifier. After each simulation run with a given parameterization, the resulting output signal was segmented by identifying the R-peaks using the Pan-Tompkins algorithm [33] and the mean signal morphology was calculated with respect to the percentage of the heart cycle.

**Figure 4:**
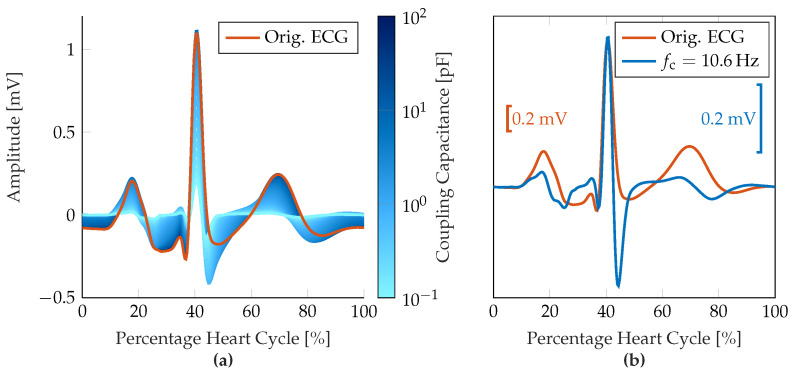
Results of the measurement chain simulations. (**a**) Resulting changes in ECG signal morphology due to input high-pass filter characteristic over varying coupling capacity $C_{\mathrm{cpl}}$. Different coupling frequencies are represented by different shades of blue from 0.1pF to 0.1nF, the original ECG signal is shown in orange. (**b**) Comparison between original ECG signal morphology and amplitude-aligned filtered signal for the input high-pass characteristic with a cut-off frequency of $f_{c}=10.6\;\mathrm{Hz}$.

The simulations were conducted for a parameterization sweep through the range of SC resistance and capacity values of the hand $R_{\mathrm{sc}}$ and $C_{\mathrm{sc}}$, respectively, and a coupling capacity $C_{\mathrm{cpl}}$ range from 0.1pF to 100pF, which corresponds to a cut-off frequency of 0.032Hz to 32Hz with $R_{\mathrm{in}}=50\;G\Omega$. Since the influence of the electrode-skin interface was found to be less significant with respect to the signal morphology (when assumed to be constant over time yet patient-individual), the simulation results were achieved with $C_{\mathrm{sc}}=10\;µF$ and $R_{\mathrm{sc}}=1\;M\Omega$. Figure 4a shows the change of the signal morphology over a varying coupling capacity $C_{\mathrm{cpl}}$ and, thus, a varying cut-off frequency $f_{c}$ of the 1st-order input high-pass filter. In Figure 4b, the signal morphology of the original ECG recording and the resulting measured ECG signal with an input high-pass filter with $C_{\mathrm{sc}}=0.3\;\mathrm{pF}$ or $f_{c}=10.6\;\mathrm{Hz}$, respectively, are compared with an aligned Q-R-amplitude.

### 2.3. Consequences for ECG Signal Evaluation

Several observations can be obtained from Figure 4 that provide information for the subsequent signal processing chain. First, the measurement signal is strongly affected by the input high-pass filter, which manifests both in amplitude as well as morphology (predominantly P-wave, S-wave, and T-wave). Especially for smaller P-wave amplitudes, the resulting waveform after high-pass filtering can be masked by noise artifacts with similar amplitude. The absence of P-waves, which is a necessary characteristic of AF, may therefore not be a reliable feature for identifying AF, especially for low signal quality-type ECG recordings. The QRS-complexes, on the other hand, still have a distinct characteristic that can be identified with standard peak detection methods, i.e., the Pan-Tompkins algorithm [33]. The T-wave tends to appear as a fully periodic sinusoidal signal at first and then turns negative as the coupling capacitance decreases. The isoelectric line is reached much earlier due to the high-pass characteristic. Therefore, important parameters for other types of cardiac arrhythmias, such as ST segment length or ST deviations, can hardly be determined reliably, so that additional diagnostic capabilities are limited.

Additionally, a high-pass filter with a comparably high cut-off frequency of $f_{c}>1\;\mathrm{Hz}$ reduces baseline wandering and thus enables valid ECG measurements with low settling time but, on the other hand, lowers the energy in the signal band resulting in a decreased SNR for constant noise components in the upper frequency bands. With respect to the ECG morphology, this results in an overall decreased R-peak amplitude and relatively amplified noise components. As far as R-peaks can be clearly distinguished from (noise) artifacts, AF can still be detected by an irregular heart rhythm or the absence of a discernible pattern of the RR intervals, respectively. According to the manual of the MyDiagnostick device, the classification algorithm is based on RR-interval dispersion, which requires reliable R-peak detection but is not dependent on P-wave visibility.

## 3. Materials and Methods

### 3.1. Pharmacy-Based AF Screening Study

**Figure 5:**
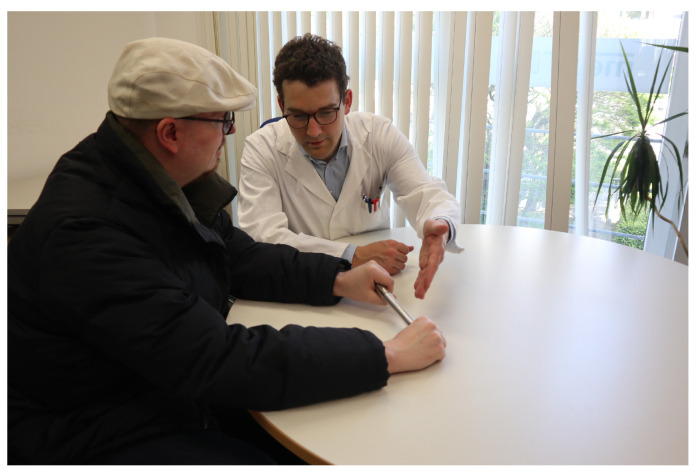
Typical measurement scenario of a potential study participant in the pharmacy. The measurement with the ECG device was performed under the guidance of trained pharmacy staff.

In a previously conducted study, a total of 7295 patients aged 65 years or older were screened for AF within a four-week period at 90 participating pharmacies in Aachen, Germany [23]. The study adhered to ethical principles and guidelines of Good Clinical Practice and was approved by the local ethics board of the University Hospital RWTH Aachen (Registration number: EK306/16, ClinicalTrials.gov: NCT03004859). The typical measurement scenario in the pharmacy is depicted in Figure 5. After exclusions due to incomplete data and lack of automated ECG analysis, 7031 data sets were eligible for further analysis for this work. 7107 recordings were automatically analyzed by the MyDiagnostick devices. The overall baseline characteristics are provided in Table 1.

**Table 1:** Baseline characteristic of pharmacy study participants with valid ECG data and resulting decision by the single-lead ECG device (normal ECG or AF detected).

Parameter	Normal ECG	AF Detected	Total
Population [1]	n=6675(93.92%)	n=432(6.08%)	n=7107
Age [years]	74±5.8	77±6.2	74±5.9
Gender [m/f]	2732/3943	245/187	2977/4130
BMI [$\mathrm{kg}/m^{2}$]	27±4.7	26±4.7	27±4.7

Trained pharmacy staff recorded a one-minute single-lead ECG using the hand-held device that provided a green or red light based on the device’s automated decision for AF detection. A study team was available for on-site or remote assistance. ECG recordings were retrospectively analyzed by ECG experts for heart rhythm and signal quality in a stepwise process, with AF defined as an episode of irregular heart rhythm for at least 30 s in absence of P-Waves. On a particular note, in this study, atrial flutter was classified as AF due to similar clinical consequences and treatment. ECG signal quality was rated as Q0: *excellent* (= 0), Q1: *good* (= 1), Q2: *poor* (= 2) or Q3: *uninterpretable* (= 3) based on the possibility of heart rhythm identification.

The clinical experts have assessed both the signal quality and the underlying nature of the ECG morphology. In case a final diagnosis for heart rhythm was not possible due to insufficient signal quality, the ECG recording was assessed as *uninterpretable*. The percentages of ratings of the clinical experts are provided in Table 2. These decisions were used as the ground truth reference for the further machine learning-based methods of quantifying the signal quality.

**Table 2:** Baseline data of signal quality assessment by clinical experts sorted by cardiological classification (Normal ECG rhythm, AF or other abnormal ECG).

	Percentage Normal ECG	Percentage AF	Percentage Other	Percentage Data Set
Signal Quality	[%] (n)	[%] (n)	[%] (n)	[%] (n)
Excellent	98.20 (817)	1.80 (15)	0 (0)	11.83 (832)
Good	95.79 (4578)	4.21 (201)	0 (0)	67.97 (4779)
Poor	88.24 (1185)	11.76 (158)	0 (0)	19.10 (1343)
Uninterpretable	0 (0)	0 (0)	100 (77)	1.10 (77)
**Total**	**93.59 (6580)**	**5.31 (374)**	**1.10 (77)**	**100 (7031)**

### 3.2. ECG Preprocessing

**Figure 6:**
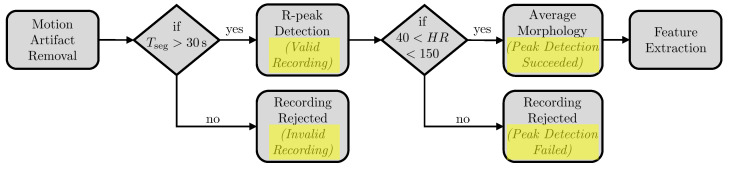
Signal processing chain for preprocessing the ECG recordings prior to feature extraction.

The signal processing for assessing the signal quality of a single ECG recording was realized in a multi-step approach. First, segments affected by motion artifacts were detected and removed by applying a custom algorithm tailored to the specific characteristics of the device used in this study. Secondly, if according to current guideline recommendations [7] the remaining continuous ECG tracing was longer than 30s, the segment was further analyzed for potential markers of signal quality. Therefore, we first identified the R-peaks to calculate the heart rate (HR) using a previously implemented adaptive method for peak detection [34]. Secondly, we extracted the average signal morphology over the heart cycle to finally calculate a set of global signal features and morphology-related features. The overall processing chain is depicted in Figure 6.

#### 3.2.1. Motion Artifact Removal

**Figure 7:**
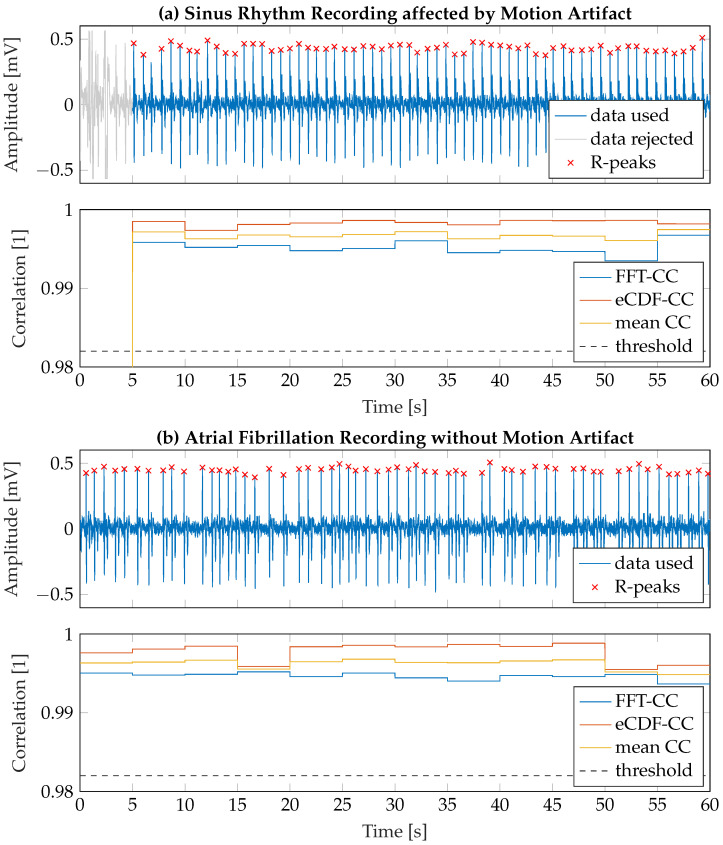
Two exemplary ECG recordings including motion artifact detection and R-peaks identified by the peak detection algorithm. (**a**) Recording of sinus rhythm affected by a motion artifact at t=0–3s. The segment affected by the motion artifact is discarded from further analysis. (**b**) Recording of a patient suffering from AF. Motion artifact detection is not affected by irregular cardiac rhythm.

For motion artifact detection and removal, the signal was processed window-based with the assumption that signal characteristics will significantly differ when affected by motion artifacts. The window length was set to 5s, such that approximately 5 consecutive heartbeats were included in a single analysis window (assuming an inter-individual average heart rate of 60bpm). In the next step, we calculated both the fast Fourier transform (FFT) and the empirical cumulative distribution function (eCDF) of each window to cover temporally independent signal characteristics of both frequency and amplitude information. Subsequently, the maximum correlation coefficients ${\mathrm{CC}}_{\max}$ were calculated for all window-based FFT and eCDF segments to determine the signal similarity to the average individual signal characteristics. Therefore, for the *i*-th window, the correlation coefficients between the FFT (or eCDF, respectively) of the *i*-th window and each other window were calculated

(2) $${\mathrm{CC}}_{i}\left[n\right]=\left\{\begin{array}{cc} \rho ({\mathrm{FFT}}_{i},\;{\mathrm{FFT}}_{j}), & \mathrm{if}\;i\neq j\\ 0, & \mathrm{if}\;i=j. \end{array}\right.$$

using Pearson’s correlation coefficient ρ and sorted in descending order represented by the vector ${\mathrm{CC}}_{i,\mathrm{sort}}$. The maximum correlation coefficient of the *i*-th window was subsequently calculated by

(3) $${\mathrm{CC}}_{\max}\left[i\right]=\frac{1}{N}\sum_{n=1}^{N}{\mathrm{CC}}_{i,\mathrm{sort}}\left[n\right].$$

with N=5. The threshold for the detection of corrupted signal windows was empirically determined as $CC_{\mathrm{thr}}=0.982$ so that, on the one hand, motion artifacts are robustly and reliably detected and, on the other hand, AF patterns are not accidentally detected as motion artifacts. When one or several motion artifacts were detected in an ECG recording, the respective segments were discarded from further analysis. If the remaining segment duration $T_{\mathrm{seg}}$ was less than 30s, the entire record was excluded from the analysis according to the current guidelines [7]. In Figure 7, two exemplary recordings and their corresponding FFT-CC and eCDF-CC values are shown. The upper graph presents a typical measurement scenario (*good* quality) in which motion artifacts occur at the beginning of the recording phase (t=0–3s) due to intentional relocating the hand positions on the stick. The lower graph shows a clean recording (*excellent* quality) of a patient suffering from AF without any significant motion artifact.

#### 3.2.2. Extraction of Average Signal Morphology

After identification of at least 30s of consecutive ECG tracings in a single recording, it was first checked for correct alignment of the device. In case that the device was held in the wrong direction, the measurement would result in the inverse Einthoven I-direction and, therefore, cannot provide reliable information with respect to comparability to other recordings of this cohort. It should be noted that the device’s approach for the detection of AF is not expected to be affected by incorrect application. Incorrect alignment can be detected based on the effective signal amplitude distribution. For correct alignment, the following assumption can be made for a signal vector s

(4) mean(s)<median(s).

If Equation (4) did not hold, the input signal was inverted for further processing. In the next step, the R-peaks were identified using a previously implemented peak detection algorithm [34] (cf. Figure 7). A recording was considered valid, if the median of the HR was within a physiologically expected range between 40bpm and 150bpm. In this way, highly noisy ECG tracings were excluded from further signal analysis, as AF detection would most likely fail when the R-peaks could not be detected reliably. The overall statistics on evaluated ECG tracings are provided in Table 3.

**Table 3:** Overall results of ECG signal preprocessing.

Signal Quality	Percentage Valid Recording	Percentage Invalid Recording	Percentage Peak Detection Succeeded	Percentage Peak Detection Failed
Excellent	100 (832)	0 (0)	99.52 (828)	0.48 (4)
Good	99.67 (4763)	0.33 (16)	99.41 (4751)	0.59 (28)
Poor	99.03 (1330)	0.97 (13)	98.96 (1329)	1.04 (14)
Uninterpretable	94.81 (73)	5.19 (4)	96.10 (74)	3.90 (3)
**Total**	**99.53 (6998)**	**0.47 (33)**	**99.30 (6982)**	**0.70 (49)**

**Figure 8:**
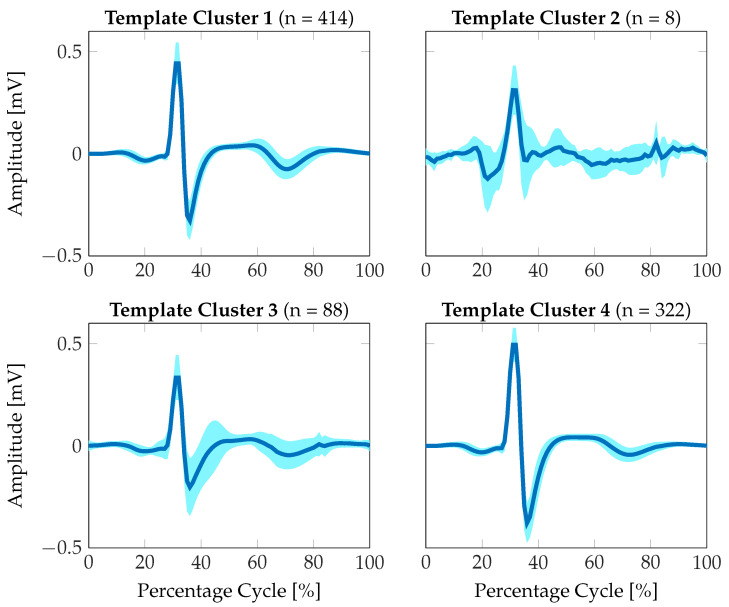
ECG heart cycle prototypes extracted from the *excellent*´signal quality category. The signal prototypes were clustered into four separate groups according to maximum signal similarity using the DTW distance. The mean signal morphology is shown in dark blue, the standard deviation is represented by the light blue area.

The ECG tracings were segmented according to the identified R-peaks and subsequently resampled to a relative percentage of the heart cycle, such that an average morphology could be determined independently of the individual HR. We then generated an ideal heart cycle prototype from the class of ECG tracings with *excellent* signal quality for quantifying the similarities of the individual signal morphologies to an ideal reference. As the morphologies within the *excellent* class also showed a certain variability, we clustered the resulting set of averaged signal morphologies according to their inter-signal distances. We calculated the dynamic time warping (DTW) distance as a cost function between two individual morphologies and identified four different clusters of optimally similar signal prototypes using the k-means clustering algorithm in MATLAB R20221b. The resulting average signal morphologies for each individual cluster for the class of *excellent* signal quality are presented in Figure 8. As can be seen, approximately 90% of the variance are covered by template clusters 1 and 4. Additionally, these clusters show minimum variance in signal morphologies. Therefore, these clusters were selected to ideally represent the signal prototypes of a heart cycle. It should be noted that the simulation results in Figure 4 also show significant similarity to the extracted signal prototype.

#### 3.2.3. Feature Extraction for Signal Quality Assessment

Different features were extracted from the raw ECG tracing as well as from the calculated average signal morphology to assess the signal quality. The signal features can be categorized into statistical features, time-domain features, frequency-domain features and morphology-related features. In total, we extracted 22 different features, which are listed and further described in Table 4. The features were calculated on a global level for each ECG recording, such that the feature vector x(s) for a single measurement was one-dimensional and temporally independent with $x\in R^{22\times 1}$.

**Table 4:** Detailed description of the set of ECG features used for the signal quality assessment.

Category	Name	Description
Statistical Features	mean	1st central moment over valid ECG signal segment
	median	Median value over valid ECG signal segment
	standard deviation	2nd central moment over valid ECG signal segment
	skewness	3rd central moment over valid ECG signal segment
	kurtosis	4th central moment over valid ECG signal segment
Time-domain Features	mean HR	Average heart rate calculated from identified RR-intervals
	SDNN	Standard deviation of RR-intervals over 1-minute recording [35]
	coverage	Segment length $T_{\mathrm{seg}}$ of valid ECG tracing (cf. Section 3.2.1)
	eCDF consistency	Mean value of eCDF correlation coefficient [36]
	Signal consistency	DTW Distance between first half and second half of ECG recording [37]
Frequency-domain Features	FFT consistency	Mean value of FFT correlation coefficient
	basSQI	Signal quality index (SQI) for ECG baseline wander according to [15]
	qrsSQI	SQI for QRS complex power according to [15]
	pliSQI	SQI for power-line interference according to [38]
	SNR	Signal-to-noise ratio according to [38]
	mean SQI	Mean SQI: (basSQI + qrsSQI + pliSQI)/3 [38]
Morphology-related Features	mean R-peak amplitude	Average R-peak amplitude from peak detection
	mean template STD	Average standard deviation from mean morphology
	Euclidean distance	Euclidean distance between mean morphology and optimal signal prototype
	Cityblock distance	One-norm between mean morphology and optimal signal prototype [39]
	DTW distance	DTW distance between mean morphology and optimal signal prototype [37]
	Correlation Coefficient	Correlation coefficient between mean morphology and optimal signal prototype

In addition to commonly used features provided by the statistical features, the time-domain and the frequency-domain features, we calculated a set of parameters that describe the similarity to the ideal ECG single-lead measurement prototype as expected by applying the device used in this study (cf. Section 3.2.2). The similarity is quantified using four metrics, which each aim at different manifestations of similarity. These are provided by the Euclidean distance, the Cityblock distance or L1 distance, the DTW distance and the correlation coefficient.

### 3.3. Regression Training

In order to automatically calculate a signal quality index from the set of features that can be extracted from every single measurement, we conducted a regression to relate the feature vectors to the clinical expert ratings. The index is denoted as *artificial intelligence-based signal quality assessment* (AISQA). The regression training was conducted using the Regression Learner toolbox in Matlab R2021b. The set of ECG recordings was separated into a training data set and a test data set with a 20% holdout with respect to the overall data set. During training, we used five-fold cross-validation to prevent overfitting. Based on the first results using different regression approaches, we focused on Gaussian process regression. The rational quadratic kernel, the Matern 5/2 kernel and the exponential kernel were used for optimizing the prediction performance.

## 4. Results

We have used the root-mean-square error (RMSE), the mean absolute error (MAE) and the Pearson correlation coefficient ρ between the clinical expert rating and the AISQA value as metrics for evaluating the SQA performance of the respective regression approach or chosen kernel. The results of the training and test phases are presented in Table 5. The combination of features through the regression formulation was projected to the arbitrarily chosen signal quality range of [0…3]. The RMSE and MAE were calculated on this basis. The balanced accuracy (BA) was calculated for the receiver operating characteristics (ROCs) with respect to the binary decision *interpretable* (*excellent*, *good* or *bad*) or *uninterpretable* signal quality.

**Table 5:** Results of the training and test phases of the Gaussian process regression using different kernel implementations. Results are presented as root-mean-square error (RMSE), mean absolute error (MAE), Pearson correlation coefficient ρ and the balanced accuracy (BA).

Phase	Kernel	RMSE [1]	MAE [1]	Pearson’s ρ [1]	BA [%]
Training/ Validation	quadratic	0.45952	0.34478	0.62269	96.37
	Matern 5/2	0.46884	0.34977	0.60161	95.12
	exponential	**0.39621**	**0.29826**	**0.75242**	**98.01**
Test	quadratic	0.46909	0.35435	0.60126	94.74
	Matern 5/2	0.47092	**0.35226**	0.59710	93.73
	exponential	**0.46800**	0.35582	**0.60365**	**94.99**

**Figure 9:**
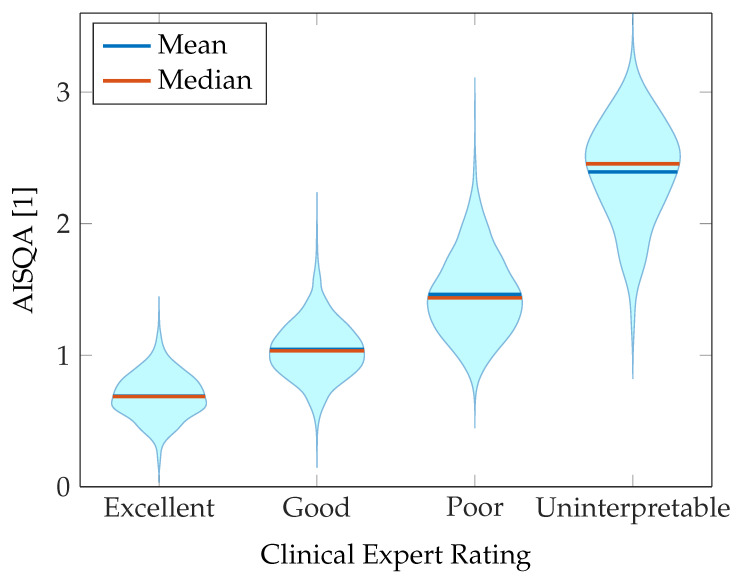
Violin plot presenting the results of the training process of mapping the input feature space to the clinical expert rating.

The training process results are additionally presented in a violin plot in Figure 9. As can be seen, the distributions concerning the reference expert rating approximately follows normal distributions. As expected, due to the heuristic rules for the assessment of signal quality by clinical experts, the four categories cannot be clearly distinguished from each other and the distributions overlap to a certain extent. However, the correlation of the calculated AISQA to the reference signal quality is evident from Figure 9. Therefore, the chosen set of features can be assumed to reflect signal characteristics that correspond to the signal quality obtained from human overread.

**Figure 10:**
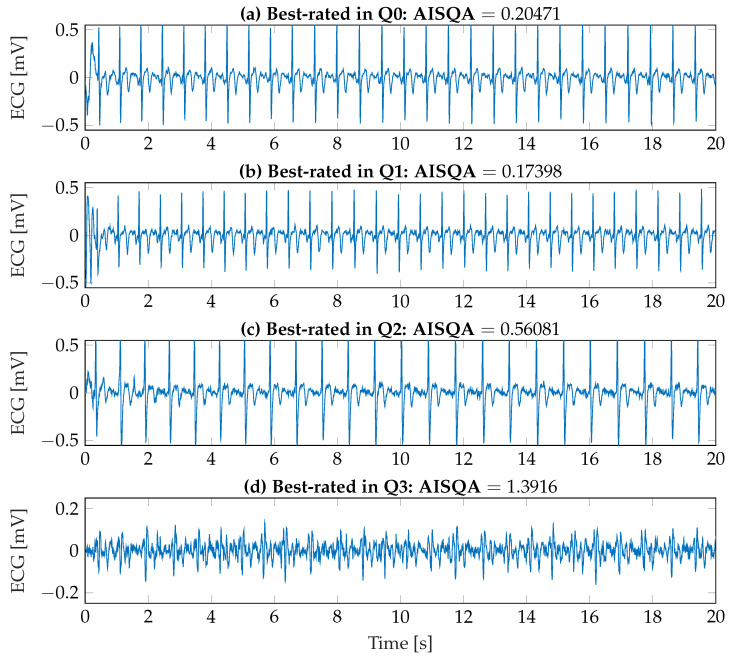
Best-rated ECG recordings according to the AISQA index from each signal quality category assessed by the clinical experts.

**Figure 11:**
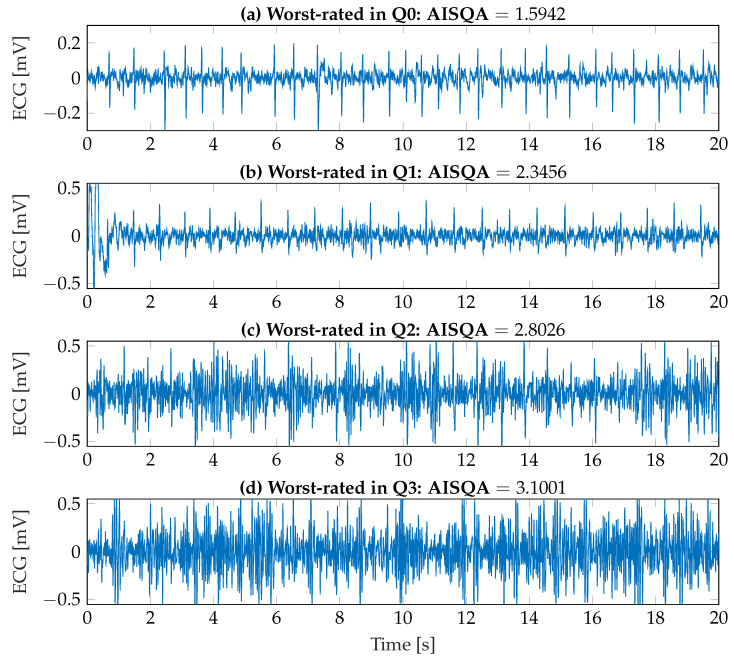
Worst-rated ECG recordings according to the AISQA index from each signal quality category as assessed by the clinical experts.

To better visualize the assessment capabilities of the implemented AISQA index, both the best-rated ECG recordings in each signal quality category and the worst-rated ECG recordings were identified and are shown in Figure 10 and Figure 11, respectively. First, as it can be observed in Figure 10a–c, the signal morphologies as introduced in Section 2.2 and Section 3.2.2 are clearly visible. Additionally, the resulting AISQA values from 0.174 to 0.561 do not significantly differ concerning the mean value of the *excellent* category of approximately 0.6. However, the best-rated ECG recording in the *uninterpretable* category with an AISQA value of 1.392 is more in the range of the *poor* signal quality. In this context, it should be noted that the single beat events are hardly detectable in Figure 10d. In contrast, the QRS complexes are still visible in Figure 11a,b. However, clearly visible P-wave activity cannot be observed, but it is beneficial and essential for the definite diagnosis of AF. The AISQA values of 1.594 and 2.346 assume a *poor* signal quality. The worst-rated recordings in the categories *poor* and *uninterpretable* are highly affected by noise, so the QRS complexes cannot be reliably detected, and the signals can be considered uninterpretable.

Finally, the correlation between falsely classified ECG recordings according to the automated decision of the device and the corresponding signal qualities was investigated. Therefore, false device classifications were identified by comparing the device’s decision to the diagnosis provided by the clinical experts. A classification was considered false if the device’s decision did not match the clinical classification. This included both false-positive and false-negative decisions and those made based on *uninterpretable* ECG recordings. In total, 3.16% (*n* =222) of the recordings were classified as possibly false. As shown in Table 6, there is a moderate correlation between the false decision of the automated AF classification and signal quality index determined by human overreading, i.e., the worse the signal quality was assessed, the higher the probability of misclassification. As can also be seen, the correlation coefficient increases slightly when AISQA is applied, suggesting that automated quality assessment can generalize the heuristic rules using the feature-based regression approach.

**Table 6:** Correlation between false decision of automated AF classification and signal quality indices (clinical expert rating vs. AISQA).

Variable	Pearson’s ρ [1] (*p*-Value [1])
Expert rating	0.2717 (p<0.001)
AISQA	0.3664 (p<0.001)

**Figure 12:**
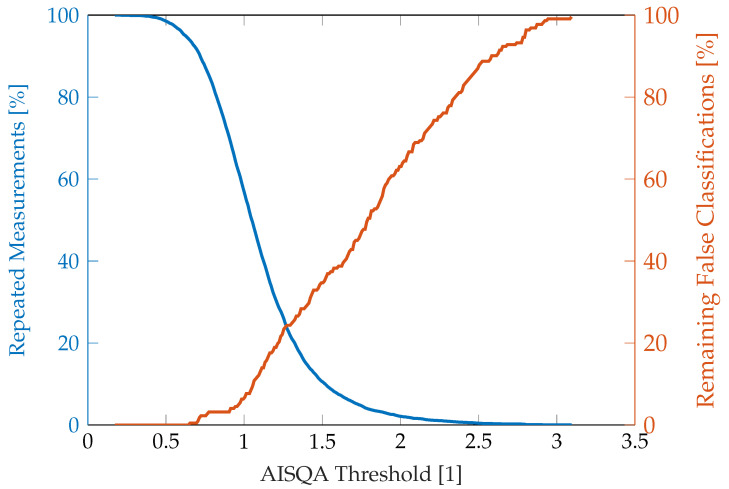
Percentages of repeated measurements and remaining false AF classification over AISQA threshold if a measurement is assumed to be repeated when the signal quality exceeds the threshold.

Figure 12 shows the number of repeated measurements over the AISQA threshold for the theoretical construct that a measurement should be repeated when a particular signal quality value is exceeded. In parallel, the percentage of remaining false classifications is shown. As can be seen, the remaining misclassifications increase approximately linearly for an AISQA value between 1 and 2.5, suggesting that misclassifications can appear almost equally distributed over the *good* and *poor* signal quality categories.

**Figure 13:**
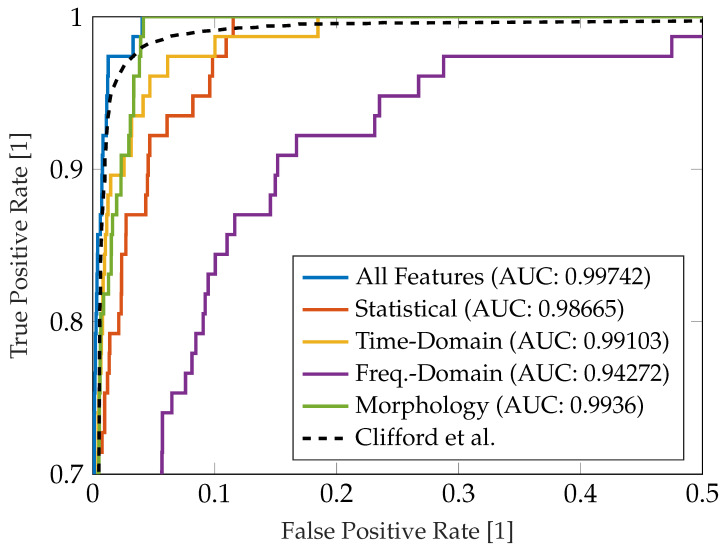
Reveiver Operating Characteristic with respect to benchmark characteristic by Clifford et al. [15] with the respective Area Under Curve (AUC) measures.

In Figure 13, the Receiver Operating Characteristic (ROC) of the implemented SQA approach with respect to the binary decision interpretable (*excellent*, *good* or *poor* signal qality) or *uninterpretable* signal quality is presented. We have evaluated the ROC for single subgroups of features (statistical, time-domain, frequency-domain and morphology-based features) as introduced in Table 4. The results are additionally compared to the performance of the overall AISQA including all features and the benchmark reference provided by Clifford et al. [15].

## 5. Discussion

Large-scale screening for cardiac arrhythmia remains a challenging task due to the specific limitations of partially unsupervised and easily applicable measurement scenarios. I.e., the measurement devices are often designed such that the patient conducts the measurement himself. This results in high requirement standards for user-independent operability to ensure reproducibility over a wide range of user expertise and environmental factors.

The device used in this study is easily applicable as the user must hold the device to start a measurement. The data is stored internally so clinical experts can analyze the recordings retrospectively. Additionally, the device includes an automated AF detection functionality that can indicate a high probability of the occurrence of AF. In the case of an abnormal R–R interval dispersion during the measurement period, the diagnosis is supposed to be substantiated by conducting an extended 12-lead ECG analysis by clinical experts. Concerning large-scale screenings, false positive decisions could increase demand for clinical diagnostic and medical resources.

The device has two metal electrodes, which avoids the need for conventional adhesive ECG electrodes. As introduced in Section 2.2, the electrode characteristics significantly influence the signal morphology, which differs from the conventional ECG due to the high-pass transfer characteristics of the passive input stage. Therefore, it should be noted that further ECG analyses, including the evaluation of P and T waves or the extraction of temporal parameters such as the PQ interval or the ST segment, require more complex signal processing to ensure reliable diagnosis. Additionally, a more extensive and automated ECG analysis would require consistently measured data. In the study presented in this paper, in some cases, the device was inadvertently held in the opposite direction, resulting in an inverse Einthoven I-lead measurement. Although this did not affect the automated AF detection of the device itself, a more detailed, automated retrospective ECG analysis with a specific focus on other cardiac abnormalities could be confounded by inconsistencies.

Overall, large-scale AF screening will clearly benefit from optimized measurement techniques and decision-making to decrease the false decision rate reducing unnecessary diagnostic workload and at the same time, increasing the acceptance and thus the applicability to a broader group of potentially high-risk subjects. This will include miniaturized integrated electronics to provide portable or wearable ECG devices and the concepts to integrate ECG measurements into everyday life situations unobtrusively. This can be achieved by ECG sticks, such as those used in this study, smart wearables, such as smartwatches or chest strap heart rate monitors, or ECG measurement technology integrated into beds, chairs, armchairs or car seats. Along with the sensor concepts, a more effective decision-making process based on novel hardware-accelerated methods, such as neuromorphic computing, could also contribute to sophisticated large-scale ECG diagnostics. This is especially concerning the increasing number of available clinical data sets for cardiac arrhythmia, which enables deep learning-based approaches for decision-making.

In addition, in the context of this study, a thorough understanding of the sources affecting the signal quality of the ECG measurements and a corresponding SQA will improve AF detection. As shown in Section 2.2, modeling and understanding the input filter stages of the ECG measurement devices help interpret the resulting signal morphology, which may appear distorted in comparison to conventional ECG measurements. Since we have identified similarities between the simulated signal morphology (cf. Figure 4) and the average ECG prototype obtained from *excellent*-quality signals (cf. Figure 8), a more detailed ECG analysis would be possible based on the a-priori knowledge of the filter characteristics.

The automated SQA implemented in this project reflected the trend of decreasing signal quality according to the clinical expert ratings (cf. Figure 9). These results suggest that the combination of extracted features is suitable for proper SQA of individual recordings. However, the distributions over the four quality categories rated by human overread are not clearly separable. In this context, it is also evident from Figure 10 and Figure 11 that the SQA by the clinical experts may be affected by subjective biases, such that the ground truth reference is subject to natural variability. Additionally, the SQAs by clinical experts were based on heuristic rules, so we cannot assume a linear relationship between the four categories. In this case, the AISQA can be considered an interpolation method that smooths clinical expert decisions based on objective signal characteristics. Although the regression approach using Gaussian processes already yielded satisfying results concerning an automated SQA, a classification approach can also be considered valid given the heuristic rules for human overread SQA.

The AISQA implemented in this project was furthermore able to increase the correlation between signal quality rating and false decisions of the ECG device. This result substantiates the assumption that the AISQA provides a valid and robust basis for assessing the ECG signal quality of this specific type of ECG device. As can be seen in Figure 12, the AISQA, however, is not perfect concerning false classifications. This graph can be further used to identify a sweet spot between the percentage of measurements to be repeated due to insufficient signal quality and remaining false classifications. It should be noted that the number of incorrect classifications and total measurements, respectively, may not be representative of such decisions. However, the AISQA needs to be further validated in a prospective study in the future. This can for example include indicating a measurement of insufficient signal quality by the device to directly repeat the recording.

Compared to previously proposed approaches for signal quality assessment of single-lead ECG recordings [15,19,20,21] our approach is based on a set of multi-domain signal features that allow for a more comprehensive analysis of the signal quality. Additionally, similar approaches often focus on spectral-based characteristics and hard thresholding, which can not directly be applied to measurement scenarios with varying coupling characteristics (cf. Section 2.2) and unspecified hardware settings. In this contribution, we have presented an approach based on multi-domain signal characteristics and a machine learning approach to assess the signal quality on a continuous scale and can be applied to any other configuration of single-lead ECG measurement devices. Consequently, the algorithm needs to be trained on a large dataset, which needs to be annotated by experts in the field to provide sufficient prediction accuracy.

Figure 13 shows the ROC curve of the different subgroups of features according to Table 4. It also compares the results of the approach implemented with a benchmark reference provided by Clifford et al., who tested their SQA method on a large dataset [15]. As can be seen, the performances of the individual subgroups highly differ. Interestingly, the feature subset that was used by Clifford et al. (cf. frequency-domain in Table 4) yielded the worst results and did not match the reference. This can be explained do to the fact that we only had a small number of recordings that were considered uninterpretable (n=77) and the heuristic classification criteria may have been different. Additionally, Clifford et al. applied their algorithm to conventional 12-lead ECG recordings. Fusing features from different domains, however, yielded satisfactory results with a comparable ROC to the reference method and is therefore considered a valid approach for assessing the signal quality of single-lead ECG data.

## 6. Conclusions

This paper demonstrates the signal deformations of a hand-held ECG diagnostic device incorporating two metal electrodes. Based on these prerequisites, we implemented an algorithm for preprocessing the ECG signal for reliable feature extraction. A regression approach was trained to map a combination of 22 features to clinical experts’ reference SQA. The process was validated using a large-scale AF screening data set with 7031 older subjects. The regression approach yielded promising results in assessing the signal quality and, therefore, reducing false classifications by repeating the measurement for exceeding a certain threshold. Future work will include further investigations on reconstructing the original ECG morphology by applying inverse filtering. Additionally, the data set will be used to implement and validate novel approaches for classifying AF with specific regard to an on-chip application.

## Data Availability

Data will be made available upon reasonable request.
